# The role of myosin 1c and myosin 1b in surfactant exocytosis

**DOI:** 10.1242/jcs.181313

**Published:** 2016-04-15

**Authors:** Nadine Kittelberger, Markus Breunig, René Martin, Hans-Joachim Knölker, Pika Miklavc

**Affiliations:** 1 Institute of General Physiology, Ulm University, Albert-Einstein Allee 11, Ulm 89081, Germany; 2 Department of Chemistry, Technische Universität Dresden, Bergstr. 66, Dresden 01069, Germany

**Keywords:** Exocytosis, Myosin 1, Surfactant secretion

## Abstract

Actin and actin-associated proteins have a pivotal effect on regulated exocytosis in secretory cells and influence pre-fusion as well as post-fusion stages of exocytosis. Actin polymerization on secretory granules during the post-fusion phase (formation of an actin coat) is especially important in cells with large secretory vesicles or poorly soluble secretions. Alveolar type II (ATII) cells secrete hydrophobic lipo-protein surfactant, which does not easily diffuse from fused vesicles. Previous work showed that compression of actin coat is necessary for surfactant extrusion. Here, we investigate the role of class 1 myosins as possible linkers between actin and membranes during exocytosis. Live-cell microscopy showed translocation of fluorescently labeled myosin 1b and myosin 1c to the secretory vesicle membrane after fusion. Myosin 1c translocation was dependent on its pleckstrin homology domain. Expression of myosin 1b and myosin 1c constructs influenced vesicle compression rate, whereas only the inhibition of myosin 1c reduced exocytosis. These findings suggest that class 1 myosins participate in several stages of ATII cell exocytosis and link actin coats to the secretory vesicle membrane to influence vesicle compression.

## Abstract (precis)

**Summary:** Myosin 1b and 1c localize to fused secretory vesicles and influence the compression of the actin coat during exocytosis.

## INTRODUCTION

Exocytosis is a pivotal mechanism for secretion of mediators, transmitters and components of the extracellular space. The fusion of a secretory vesicle with the plasma membrane depends on a conserved group of proteins, which mediate the opening of the fusion pore (Fang and Lindau, 2014; Jahn and Fasshauer, 2012; Rizo and Südhof, 2012). Exocytosis is also influenced by actin and actin-associated proteins, which have inhibitory as well as stimulating roles in the pre-fusion and post-fusion phase of exocytosis (Eitzen, 2003; Malacombe et al., 2006; Papadopulos et al., 2013; Porat-Shliom et al., 2013). Although cortical actin network presents a barrier for vesicle fusion with the plasma membrane (Giner et al., 2005), actin fibers and associated motor proteins can facilitate vesicle transport to the site of exocytosis (Rojo Pulido et al., 2011). In addition, recent data obtained on cells with large secretory vesicles or poorly soluble secretions indicate that formation of the actin coat on fused vesicles has an important role in the post-fusion phase of exocytosis. Actin coat promotes compensatory endocytosis in oocytes (Sokac et al., 2003), stabilizes secretory vesicles in pancreatic acinar cells (Nemoto et al., 2004; Thorn, 2009) and provides the force for active content extrusion in endothelial cells (Nightingale et al., 2011, 2012), salivary gland cells (Masedunskas et al., 2011) and ATII cells (Miklavc et al., 2009, 2012, 2015). Several groups of myosin motor proteins associate with actin coats and influence its compression or organization (Masedunskas et al., 2011; Miklavc et al., 2012; Sokac et al., 2006; Yu and Bement, 2007).

Class 1 myosins function as linkers between membranes and actin cytoskeleton in several cellular processes. They have an N-terminal actin- and nucleotide-binding head domain, a calmodulin-binding neck region and a C-terminal membrane-binding tail domain (Barylko et al., 2005; McConnell and Tyska, 2010). The tail domain contains a pleckstrin homology (PH) domain, which enables direct association between the myosin tail and acidic membrane lipids such as phosphatidyl inositol (4,5)-bisphosphate (PIP_2_) (Hokanson et al., 2006). Myosin 1 function is regulated by intracellular Ca^2+^, which binds to calmodulin and causes its dissociation from the neck domain (Lin et al., 2005; Manceva et al., 2007; Swanljung-Collins and Collins, 1991; Zhu et al., 1996). Of the eight known myosin 1 isoforms in vertebrates, myosin 1a, 1c and 1b (Myo1a, Myo1c and Myo1b, respectively) are the best characterized (Greenberg and Ostap, 2013; McConnell and Tyska, 2010). Myo1a is expressed only in enterocytes, where it links actin bundles to the plasma membrane of brush border microvilli (Crawley et al., 2014). Myo1c influences numerous cellular processes, reaching from hair cell adaptation (Gillespie and Cyr, 2004; Stauffer et al., 2005), to regulation of gene expression (Sarshad et al., 2013), G-actin transport during cell migration (Fan et al., 2012) and intracellular membrane trafficking (Bose et al., 2002, 2004; Brandstaetter et al., 2012; Chen et al., 2007; Sokac et al., 2006). Myo1c promotes insulin-dependent exocytosis of GLUT4-containing vesicles in muscle cells and adipocytes (Bose et al., 2002, 2004; Chen et al., 2007) and delivery of lipid-raft-associated proteins to the cell surface in HeLa cells (Brandstaetter et al., 2012). In *Xenopus* oocytes, Myo1c links the actin coat to fused cortical granules and transduces force generated by the actin coat to compress the vesicle membrane (Sokac et al., 2006). In contrast, Myo1b colocalizes with endosomes (Raposo et al., 1999; Salas-Cortes et al., 2005) as well as with the plasma membrane (Komaba and Coluccio, 2010) and plays a role in generation of tubules from the Golgi network (Almeida et al., 2011; Coudrier and Almeida, 2011) and in ephrin signaling (Prospéri et al., 2015). Although Myo1c and Myo1b share structural similarities, their biophysical properties differ. Myo1c can generate force over a range of loads and has therefore been suggested to play a role as a transport protein (Greenberg and Ostap, 2013; Greenberg et al., 2012). In contrast, Myo1b is extremely sensitive to load and more likely functions as a force-sensitive anchor (Greenberg and Ostap, 2013; Laakso et al., 2008; Shuman et al., 2014).

Here, we investigate the localization and function of Myo1b and Myo1c during exocytosis of surfactant-containing secretory granules (lamellar bodies) in ATII cells. Surfactant is a hydrophobic material made of lipids and proteins, which inserts in the alveolar lining fluid to reduce surface tension and enable inspiration (Dietl and Haller, 2005; Dietl et al., 2004). The hydrophobicity of surfactant precludes simple diffusion from the fused vesicle and recent studies have shown that actin coat formation on fused vesicles and its compression are pivotal for surfactant extrusion (Miklavc et al., 2012, 2015). In this study, we show that both isoforms, Myo1c and Myo1b, translocate to fused lamellar bodies. However, their kinetics of translocation were strikingly different. Slow recruitment of Myo1b to the vesicle membrane was likely due to an inhibitory effect of the motor activity in the head domain, whereas the translocation of Myo1c depended on the intact PH domain in the tail region. Translocation of both isoforms was sensitive to Ca^2+^. Myo1c inhibition reduced exocytosis and slowed down actin coat compression. In contrast, inactivation of the motor domain of Myo1b enhanced the post-fusion vesicle compression.

## RESULTS

### Endogenous expression of Myo1 isoforms in ATII cells

**Fig. 1.**
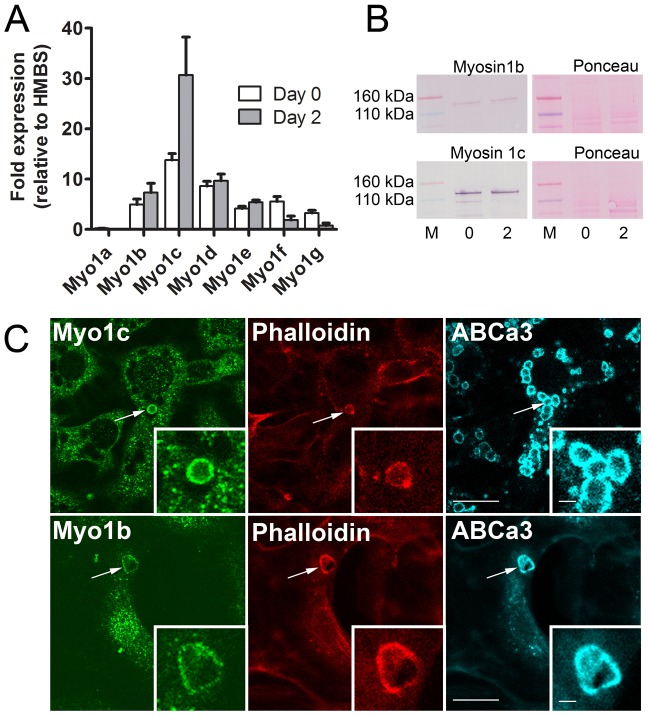
**Expression and localization of Myo1b and Myo1c in ATII cells.** (A) Semi-quantitative RT-PCR showed that Myo1b and Myo1c are among the highest expressed Myo1 isoforms in ATII cells. Data (mean±s.e.m.) obtained from three cell isolations and three experiments per isolation are shown relative to the expression of the housekeeping gene *HMBS*. Day 0, freshly isolated cells; Day 2, after 2 days in culture. (B) Western blot of Myo1b and Myo1c from freshly isolated ATII cells and ATII cells after 2 days of culture (0 and 2, respectively) to demonstrate expression of Myo1b and Myo1c in ATII cells. Ponceau S staining was used to control for equal protein loading (M, marker). (C) Colocalization of anti Myo1b and Myo1c staining with fused secretory vesicles marked by ABCa3 and phalloidin immunostaining. The ABCa3 transporter is a marker for secretory vesicle membrane in ATII cells. After ATP stimulation, some vesicles fused with the plasma membrane and acquired an actin coat, which was labeled by phalloidin staining (arrow). Scale bars: 10 µm. Insets show the enlarged view of the fused lamellar bodies. Scale bars: 1 µm.

To investigate the role of myosin 1 for ATII cell exocytosis, we first measured the relative expression of myosin 1 isoforms by performing semi-quantitative RT-PCR (Fig. 1A). Myo1c, Myo1b and Myo1d had the highest expression rate in freshly isolated ATII cells as well as after 2 days of culture, whereas the lowest expression was detected for Myo1a and Myo1g. In this work, we focus on the localization and role of Myo1b and Myo1c during exocytosis in ATII cells as the biophysical properties of both isoforms are well-characterized and commercial antibodies for immunostaining experiments on rat cells are available. In addition, Myo1c has already been described to participate in exocytosis (Bose et al., 2002; Sokac et al., 2006). Myo1b and Myo1c could be detected in ATII cells in western blot experiments (Fig. 1B) and on the membrane of fused lamellar bodies in immunostaining experiments, where the lamellar body membrane was labeled by immunostaining of the ABCa3 lipid transporter, and fused vesicles were differentiated from non-fused vesicles by the presence of actin coats (phalloidin staining) (Fig. 1C).

### Myo1b and Myo1c translocation to the limiting membrane of secretory vesicles

**Fig. 2.**
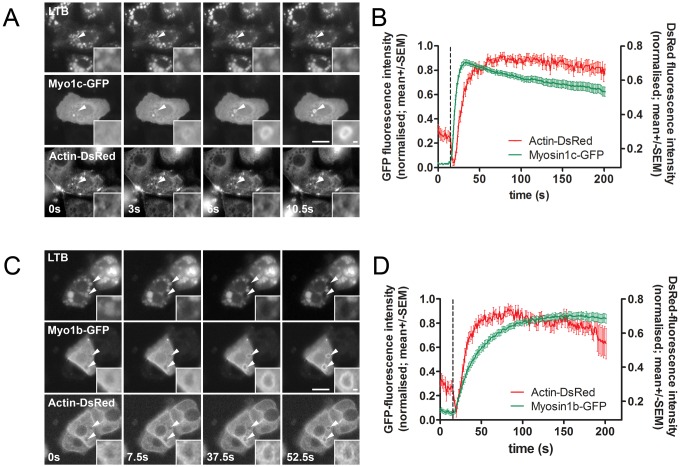
**Translocation of Myo1b–GFP and Myo1c–GFP to fused secretory vesicles.** (A) ATII cells were co-transfected with Myo1c–GFP and actin–DsRed and stained with LTB. Myo1c–GFP translocated to secretory vesicles after fusion. Arrowheads indicate fusing vesicles and time=0 indicates the last frame before fusion. Scale bar: 10 µm. The time of fusion pore opening was determined by LTB fluorescence decrease (upper row). Insets show an enlarged view of the fused vesicle. Scale bar: 1 µm. (B) Mean±s.e.m. of the Myo1c–GFP and actin–DsRed fluorescence change on fusing lamellar bodies (*n*=31). Myo1c–GFP translocated to fused vesicles before actin polymerization. The dashed line indicates the last frame before the fusion pore opening, determined by LTB fluorescence decrease. (C) ATII cells were co-transfected with Myo1b–GFP and actin–DsRed and stained with LTB. Myo1b–GFP translocated to secretory vesicles after fusion. Arrowheads point at fusing lamellar bodies and time=0 indicates the last frame before the fusion pore opening. Scale bar: 10 µm. The time of fusion pore opening was determined by LTB fluorescence decrease (upper row). Insets show an enlarged view of one fusing vesicle. Scale bar: 1 µm. (D) Mean±s.e.m. of the Myo1b–GFP and actin–DsRed fluorescence change on the fusing lamellar bodies (*n*=42). The kinetics of Myo1b–GFP translocation was slower than actin polymerization and also slower than Myo1c translocation (B). The dashed line indicates the last frame before the fusion pore opening.

We investigated the kinetics of Myo1b and Myo1c translocation to fused lamellar bodies by transfecting ATII cells with either Myo1b–GFP or Myo1c–GFP. The time point of lamellar body fusion was determined with LysoTracker Blue (LTB), which accumulates in lamellar bodies and diffuses in the extracellular space during exocytosis, resulting in a rapid decrease of fluorescent vesicle staining (Haller et al., 1998; Miklavc et al., 2012). Cells were co-transfected with actin–DsRed to detect actin coat formation on fused vesicles. Myo1b–GFP and Myo1c–GFP both translocated to the lamellar body membrane after fusion (Fig. 2). Translocation of Myo1c–GFP to the vesicle membrane was significantly faster than the formation of the actin coat (the half-time of Myo1c–GFP fluorescence increase on fused lamellar bodies calculated by a one-phase association fit was 5.13±0.9 s, *n*=31 and the half-time of actin polymerization was 9.90±1.26 s, *n*=36; *P*=0.003; mean±s.e.m.; Fig. 2A,B). In contrast, Myo1b–GFP translocation to the fused vesicles was significantly slower than actin coat formation (the half-time of Myo1b–GFP fluorescence increase on fused lamellar bodies was 27.65±3.14 s, *n*=23; *P*<0.0001; Fig. 2C,D).

**Fig. 3.**
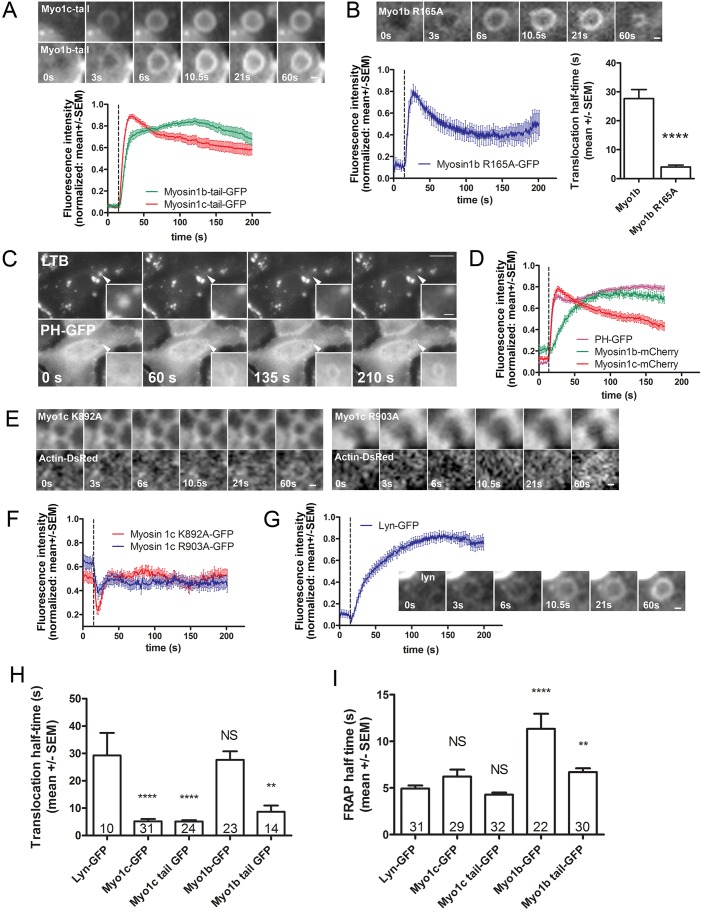
**Kinetics of Myo1b and Myo1c translocation to fused vesicles.** (A) The image series shows single fusing vesicles in Myo1b-tail–GFP- or Myo1c-tail–GFP-transfected ATII cells. The Myo1b-tail–GFP and Myo1c-tail–GFP translocated to fused secretory vesicles. Time=0 indicates the last frame before the fusion pore opening. Scale bar: 1 µm. The graph shows the mean±s.e.m. of the Myo1b-tail–GFP and Myo1c-tail–GFP fluorescence increase on fused lamellar bodies (*n*=28 and 38, respectively). Both tail domains translocated to the fused secretory vesicles with similar kinetics. The dashed line indicates the last frame before the fusion pore opening. (B) Myo1b-R165A–GFP translocated to fused vesicles with faster kinetics than Myo1b–GFP (see Fig. 2D). The image series shows single fusing vesicle in ATII cells transfected with Myo1b-R165A–GFP. Scale bar: 1 µm. The graphs show the mean±s.e.m. of the Myo1b-R165A–GFP fluorescence change on the site of the fused lamellar bodies (*n*=18, left) and the comparison of the half-times of Myo1b–GFP and Myo1b-R165A–GFP fluorescence increase on fused vesicles (*n*=23 and 10, respectively; right). The dashed line on the graph and time=0 s on the image series indicate the last frame before the fusion pore opening. The half-time of Myo1b-R165A–GFP was significantly shorter compared to full-length Myo1b–GFP. ****P<0.0001 (two-tailed Mann–Whitney test). (C) ATII cells transfected with PH–GFP and stained with LTB. The arrowhead points at the fusing lamellar body and the insets show an enlarged view of the fused vesicle. PH–GFP translocated to secretory vesicles after fusion (bottom row). The time of fusion pore opening was determined by LTB fluorescence decrease (upper row). Time=0 indicates the last frame before the fusion pore opening. Scale bars: 10 µm (image); 1 µm (inset). (D) Mean±s.e.m. of the PH–GFP, Myo1b–mCherry and Myo1c–mCherry fluorescence increase on the fused lamellar bodies (*n*=97, 36 and 61, respectively). The dashed line indicates the last frame before the fusion pore opening, determined by LTB fluorescence decrease. Note the similar kinetics of lamellar body localization in PH–GFP and Myo1c–mCherry and the slower translocation of Myo1b–mCherry. (E) Single fusing vesicles in ATII cells transfected with GFP-coupled Myo1c constructs with a PH-domain mutation R903A or K892A and co-transfected with actin–DsRed. These constructs had cytoplasmic localization and did not translocate to fused vesicles after fusion. The GFP fluorescence (above) and DsRed fluorescence (below) are shown for each vesicle. Time=0 indicates the last frame before the fusion pore opening. Scale bar: 1 µm. (F) Mean±s.e.m. of the Myo1c-K892A–GFP and Myo1c-R903A–GFP fluorescence change on the site of the fused lamellar bodies (*n*=34 and 30, respectively). No translocation of Myo1c-K892A–GFP and Myo1c-R903A–GFP to secretory vesicles could be observed after fusion. Fusion pore opening is indicated by the dashed line. (G) Mean±s.e.m. of the Lyn–GFP fluorescence increase on fused lamellar bodies (n=20) and an image sequence of a single fusing vesicle in a Lyn–GFP-transfected ATII cell. Lyn–GFP translocated to fused vesicles with relatively slow kinetics compared to Myo1c–GFP or PH–GFP. The dashed line on the graph and *t*=0 s on the image sequence indicate the last frame before the fusion pore opening. Scale bar: 1 µm. (H) Translocation half-times of GFP-tagged Myo1b and Myo1c constructs compared to Lyn–GFP. The translocation of Myo1b–GFP to the membrane of fused vesicles was not significantly different from Lyn–GFP, whereas the translocation of other tested constructs was significantly faster (the *n* values are as given on the columns and represent the number of vesicles). **P<0.01; ****P<0.0001; NS, not significant (two-tailed Mann–Whitney test). (I) Half times of fluorescence recovery after photobleaching for the same constructs shown on H. The bleaching area (diameter=4 µm) was chosen on the edge of the cell and fluorescence recovery was measured in 0.5 s intervals. The FRAP half-time of Lyn–GFP was not significantly different from that of the Myo1c–GFP and Myo1c-tail–GFP constructs, whereas the FRAP half-time was markedly prolonged in Myo1b–GFP (the *n* values are as given on the columns and represent the number of cells where FRAP was performed). **P<0.01; ****P<0.0001; NS, not significant (two-tailed Mann–Whitney test).

To investigate the reasons for the striking difference in the translocation kinetics of Myo1c and Myo1b, we transfected the cells with GFP-coupled constructs containing only the membrane-binding tail domains of Myo1b or Myo1c (Fig. 3A). Because the expression levels could influence the translocation kinetics, we compared the GFP fluorescence intensity of cells expressing different Myo1 constructs by performing either fluorescence microscopy measurement on single cells or a plate reader assay. There was no significant difference in GFP fluorescence between the Myo1c or Myo1b wild-type (wt) and tail constructs (Fig. S1). The half-time of Myo1c-tail–GFP translocation to the vesicle membrane (5.11±0.49 s, *n*=24; mean±s.e.m.) was not significantly different from that of full-length Myo1c, suggesting that the fast translocation of the full-length Myo1c reflects the association of its tail domain with the vesicle membrane. In contrast, the half-time of Myo1b-tail–GFP fluorescence increase (8.67±2.27 s, *n*=14) was significantly shorter compared to the full-length Myo1b–GFP (*P*<0.0001) and not significantly different from Myo1c–GFP or Myo1c-tail–GFP. This observation indicates that the head domain might be responsible for the slow translocation of full-length Myo1b to fused vesicles. To investigate whether the slow translocation was due to the motor activity of the Myo1b head domain, we inserted a R165A mutation into Myo1b to block ATP hydrolysis and inactivate Myo1b motor activity (Komaba and Coluccio, 2010; Shimada et al., 1997). The Myo1b-R165A–GFP mutant translocated to the fused lamellar bodies significantly faster than full-length Myo1b–GFP (Fig. 3B). The half-time of Myo1b-R165A–GFP fluorescence intensity increase on fused lamellar bodies (3.98±0.71 s; *n*=10) was significantly shorter than the half-time of wt Myo1b–GFP translocation (*P*<0.0001, Mann–Whitney test; Fig. 3B) and not significantly different from that of the Myo1b-tail–GFP. Myo1b R165A translocation was mostly transient and only detectable on 75% of fused vesicles (*n*=24; Fig. 3B), in contrast to wt Myo1b–GFP, which was recruited to vesicle membrane in all observed fusion events.

Both Myo1 isoforms, 1b and 1c, have been described to contain a PIP_2_-binding PH domain in their tail region (Hokanson et al., 2006; Komaba and Coluccio, 2010), therefore we investigated the possibility that Myo1b and Myo1c were recruited to fused lamellar bodies through PIP_2_ in the lamellar body membrane. To image PIP_2_ localization in living cells we used a GFP-labeled PH domain of phospholipase Cδ (Czech, 2000; Várnai and Balla, 2007). PH-GFP was rapidly recruited to the lamellar body membrane after fusion (half-time 3.65±0.49 s, *n*=22; Fig. 3C) indicating that PIP_2_ was either translocated to or formed on the fused lamellar bodies. To detect PIP_2_ together with Myo1b or Myo1c during lamellar body exocytosis in the same cells, we co-transfected the cells with PH–GFP and either Myo1c–mCherry or Myo1b–mCherry (Fig. 3D). The half-time of PH–GFP fluorescence increase on fused vesicles was not significantly different from that of Myo1c–mCherry and significantly shorter than that of the Myo1b–mCherry fluorescence increase (*P*<0.001). The remarkable similarity between the translocation kinetics of PH–GFP and Myo1c–mCherry suggests that the PH domain on the tail of Myo1c might determine Myo1c translocation. To investigate this possibility, we transfected ATII cells with Myo1c-K892A–GFP and Myo1c-R903A–GFP constructs, where the mutations in the PH domain of Myo1c tail region prevent PIP_2_ binding (Hokanson et al., 2006). Both mutation-containing constructs localized to the cell cytoplasm and did not translocate to the lamellar body membrane after exocytosis (Fig. 3E), indicating the importance of the PH domain for Myo1c translocation to the fused vesicles.

Irrespective of the lipids required for Myo1b–GFP and Myo1c–GFP attachment to the vesicle membrane, these isoforms could translocate to the vesicle membrane either by lateral diffusion from the plasma membrane into the vesicle membrane or they could be recruited from the cytosol. To investigate which possibility was more likely, we compared the recruitment of Myo1b–GFP and Myo1c–GFP to the fused vesicles with the recruitment of a GFP-coupled Lyn-kinase membrane anchor domain. Lyn–GFP inserts into the inner leaflet of the plasma membrane with acyl anchors and translocates to the membrane of fused vesicles by lateral diffusion (Fig. 3G; Miklavc et al., 2012). The half-time of wt Myo1b–GFP translocation to the lamellar body membrane was not significantly different from Lyn–GFP (half-time 29.29±8.25 s, *n*=10; mean±s.e.m.), whereas Myo1c and Myo1c tail constructs translocated to the vesicle membrane significantly faster than Lyn–GFP (*P*<0.0001, Mann–Whitney test; Fig. 3H). To establish whether the reason for this discrepancy lies in the different mobility of the constructs in the plane of the plasma membrane, we performed FRAP experiments. The half-time of fluorescence recovery for Lyn–GFP (4.94±0.34 s, *n*=31) was not significantly different from that of Myo1c–GFP (6.22±0.75 s, *n*=29) or Myo1c-tail–GFP (4.28±0.22 s, *n*=32), suggesting that their mobility in the plasma membrane might be similar (Fig. 3I). In contrast, the fluorescence recovery half-time of Myo1b–GFP (11.33±1.63 s, *n*=22) and Myo1b-tail–GFP (6.7±0.41 s, *n*=30) were significantly longer than that of Lyn–GFP (*P*<0.0001 and *P*=0.002, respectively; Mann–Whitney test).

### Ca^2+^ dependence of Myo1b and Myo1c translocation to fused vesicles

**Fig. 4.**
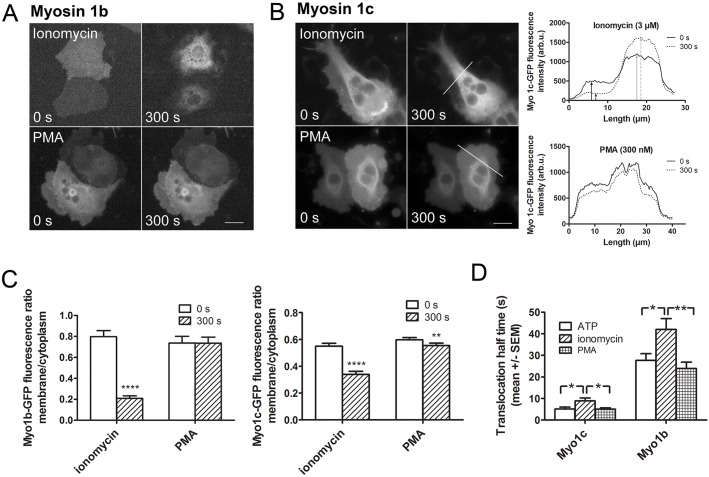
**Ca^2+^ dependency of Myo1b and Myo1c translocation.** (A) Myo1b–GFP-transfected ATII cells before (time=0 s) and after (300 s) stimulation with either ionomycin (upper row) or PMA (bottom row). A change in Myo1b localization was only observed after ionomycin stimulation. Scale bar: 10 µm. (B) Myo1c–GFP-transfected ATII cells (left) before (time=0 s) and after (300 s) stimulation with either ionomycin (upper row) or PMA (bottom row). Scale bar: 10 µm. The lines on the right side indicate the location of the line scan measurement. Line scans were used to calculate the ratio between the fluorescence intensity of the edge of the cell (black arrows) and the center of the cell (gray arrows), before (solid line) and 300 s after stimulation (dashed line). (C) Comparison of the fluorescence ratio (mean±s.e.m.) between the cell membrane and the cell cytoplasm for ionomycin or PMA stimulation in ATII cells transfected with either Myo1b–GFP (left graph) or Myo1c–GFP (right graph). The fluorescence ratio between the membrane and the cytoplasm of Myo1b–GFP and Myo1c–GFP decreased significantly after ionomycin stimulation, whereas PMA stimulation only affected Myo1c localization. *n*=29 and 25 cells were transfected with Myo1b–GFP, and 32 and 33 cells with Myo1c–GFP for ionomycin and PMA stimulation, respectively. **P<0.01, ****P<0.0001 (two-tailed Wilcoxon matched-pairs signed rank test). (D) Half-times (mean±s.e.m.) of Myo1c–GFP and Myo1b–GFP translocation to fused vesicles after cell stimulation with either ATP, ionomycin or PMA. After ionomycin stimulation the translocation of both myosin isoforms, Myo1b–GFP and Myo1c–GFP was significantly slower than after PMA or ATP stimulation. *n*=31, 14 and 9 vesicles in Myo1c–GFP-transfected cells, and 23, 9 and 8 vesicles in Myo1b–GFP-transfected cells for ATP, ionomycin and PMA stimulation, respectively. *P<0.05; **P<0.01 (two-tailed *t*-test).

Changes in cytoplasmic Ca^2+^ concentration influence Myo1 motor activity (Greenberg and Ostap, 2013; McConnell and Tyska, 2010) and can also affect their binding to membranes directly (Tang et al., 2002) or by changing membrane properties (Hokanson and Ostap, 2006). To investigate the influence of Ca^2+^ ions on Myo1b and Myo1c localization during ATII cell exocytosis, we applied the Ca^2+^ ionophore ionomycin or phorbol-12-myristate-13-acetate (PMA) to cells transfected with either Myo1b–GFP or Myo1c–GFP. Ionomycin causes a strong Ca^2+^ influx from the intracellular space, whereas PMA stimulates lamellar body exocytosis by directly activating protein kinase C and was used as control to elicit fusions without an increase in intracellular Ca^2+^ concentration (Frick et al., 2001). First, we determined whether the association of Myo1b–GFP and Myo1c–GFP with the plasma membrane is Ca^2+^ dependent. We calculated the association of both constructs to the plasma membrane as a ratio between the fluorescence intensity at the edge of the cell and in the middle of the cell before and 300 s after ionomycin or PMA stimulation (Fig. 4A,B) (Cai et al., 2013). The localization of Myo1b and Myo1c to the plasma membrane was confirmed by co-staining with membrane dye DiI (Fig. S2). Ionomycin caused a significant decrease in the fluorescence ratio for both myosin isoforms (from 0.55±0.02 to 0.34±0.02, *n*=32, *P*<0.0001 in Myo1c and from 0.8±0.06 to 0.21±0.02, *n*=29, *P*<0.0001 in Myo1b; mean±s.e.m.), which reflected the translocation from the plasma membrane to the cytosol upon Ca^2+^ elevation (Fig. 4C). PMA induced a small but significant decrease in membrane-to-cytosol ratio in Myo1c-transfected cells (0.6±0.02 to 0.55±0.02, *n*=33, *P*=0.001), whereas the difference in Myo1b-transfected cells was not significant (0.74±0.06 before stimulation to 0.74±0.06 after stimulation, *n*=25; Fig. 4C). The significance was calculated with the Wilcoxon matched-pairs signed rank test.

Because Ca^2+^ had such a profound influence on Myo1c–GFP and Myo1b–GFP association with the plasma membrane, we tested whether there is a difference in the post-fusion translocation of these constructs to the vesicle membrane. We measured translocation half-times after ionomycin or PMA stimulation, and compared those with values after ATP stimulation, which is a physiological stimulus for lamellar body exocytosis. In ATII cells, ATP induces a transient general intracellular Ca^2+^ increase by stimulating Ca^2+^ release from the intracellular stores (Rooney, 2001), followed by a local perivesicular Ca^2+^ increase around fused vesicles (Miklavc et al., 2010, 2011). The half-time of Myo1c–GFP translocation to vesicle membrane after ionomycin stimulation was 8.90±1.32 s (*n*=14), which was significantly longer than after PMA stimulation (5.10±0.60 s, *n*=9; *P*=0.04) or after ATP stimulation (5.13±0.9 s, *n*=31; *P*=0.02; Fig. 4D). A similar pattern was observed in Myo1b–GFP-transfected cells, where the translocation half-time after ionomycin stimulation (42.03±5.04 s, *n*=9) was significantly longer than the half-time of translocation after PMA stimulation (23.95±2.97 s, *n*=8; *P*=0.009) and after ATP stimulation (27.65±3.14 s, *n*=23; *P*=0.02). The differences between ATP and PMA stimulation were not significant for both constructs (Fig. 4D).

### Myo1 influence on exocytosis in ATII cells

**Fig. 5.**
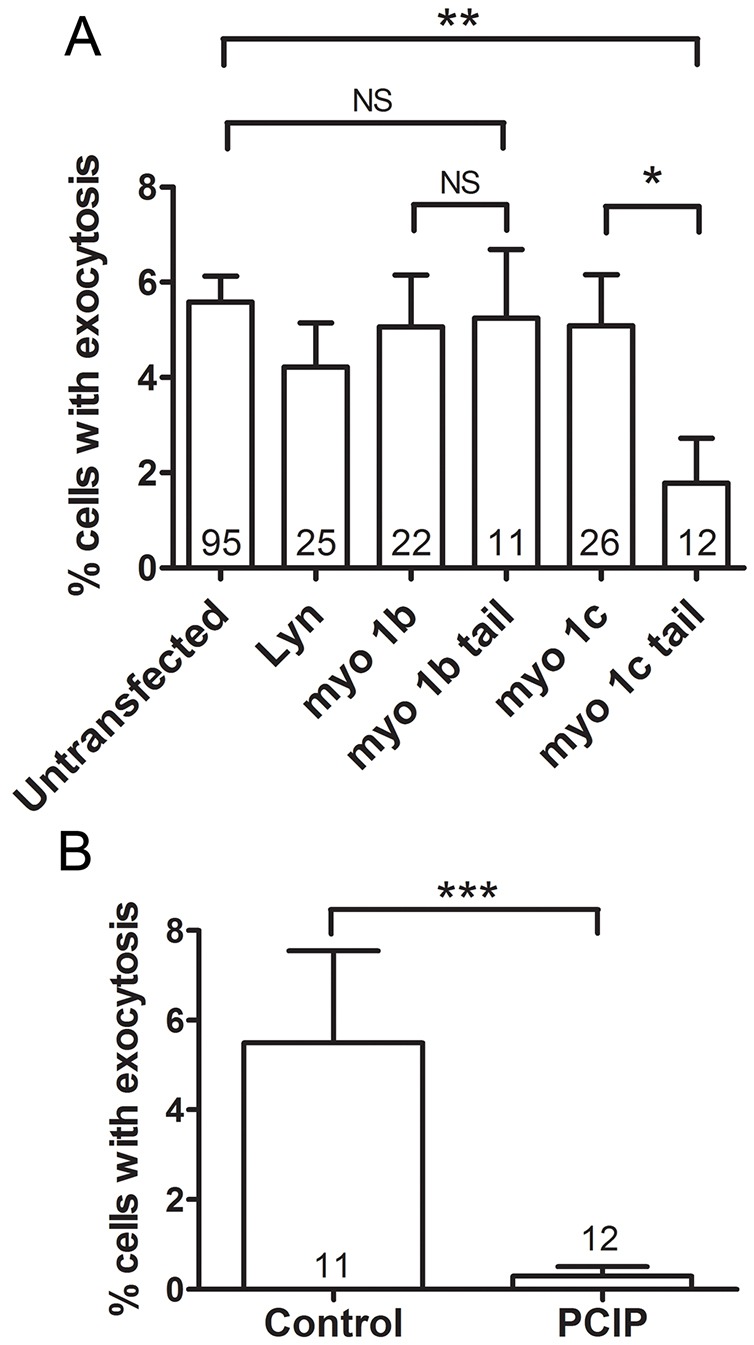
**The influence of Myo1c and Myo1b on exocytosis.** (A) The proportion (mean±s.e.m.) of cells responding to ATP stimulation with exocytosis in untransfected ATII cells and in cells transfected with Lyn–GFP control construct or Myo1b or Myo1c constructs. The percentage of cells showing exocytosis was significantly reduced in cells transfected with Myo1c tail-GFP compared to untransfected cells and full-length Myo1c-GFP. Transfection with Myo1b tail-GFP did not significantly change the proportion of cells with exocytosis. The numbers in the columns indicate the number of independent experiments, up to four time-lapse image sequences were analyzed for each experiment, up to 56 cells were analyzed for each image sequence. *P<0.05; **P<0.01; NS, not significant (two-tailed Mann–Whitney test). (B) Incubation of ATII cells with 10 µM PClP inhibitor for 2 h significantly decreased the percentage of cells responding to ATP stimulation with exocytosis compared to untreated cells. ***P<0.001 (two-tailed Mann–Whitney test). The numbers in the columns indicate the number of independent experiments, up to four time-lapse image sequences were analyzed for each experiment, and up to 36 cells were analyzed for each image sequence.

After we researched the possible causes for Myo1b and Myo1c translocation to the fused lamellar bodies, we were interested in their function for lamellar body exocytosis. It has already been described that inhibition of Myo1c has a negative effect on insulin-dependent exocytosis of GLUT4-containing vesicles in adipocytes (Bose et al., 2002, 2004) and skeletal muscle cells (Toyoda et al., 2011). We tested the influence of Myo1c and Myo1b on exocytosis in ATII cells by expressing the dominant-negative Myo1b tail or Myo1c tail domain. The effect on exocytosis was determined by counting the number of cells, which responded to ATP stimulation with at least one fusion event (Fig. 5A). Transfection with Myo1c-tail–GFP reduced the proportion of responding cells (1.78±0.94%, *n*=12 independent experiments; mean±s.e.m.) compared to untransfected cells (5.58±0.54%, *n*=95 independent experiments; *P*=0.002), cells transfected with the wt Myo1c–GFP (5.08±1.07%, *n*=26 independent experiments; *P*=0.04), or cells transfected with control construct Lyn–GFP (4.21±0.92%, *n*=25 independent experiments; *P*=0.08). In contrast, transfection with the Myo1b-tail–GFP did not affect exocytosis (5.24±1.44%, *n*=11 independent experiments). Up to four time-lapse image sequences were analyzed for each experiment, up to 56 cells were analyzed for each image sequence. A Mann–Whitney test was used for statistics.

In addition to genetic inhibition, we used pharmacological inhibition with pentachloropseudilin (PClP), an allosteric inhibitor specific for class 1 myosins (Chinthalapudi et al., 2011; Martin et al., 2009). The percentage of ATII cells responding after 10 µM PClP treatment (0.29±0.2%, *n*=12 independent experiments) was significantly lower than the percentage of responding cells in untreated control cells (5.49±2%, *n*=11 independent experiments; *P=*0.0006, Mann–Whitney test; Fig. 5B). Up to four time-lapse image sequences were analyzed for each experiment.

### Myo1 coupling between actin coat and vesicle membrane

**Fig. 6.**
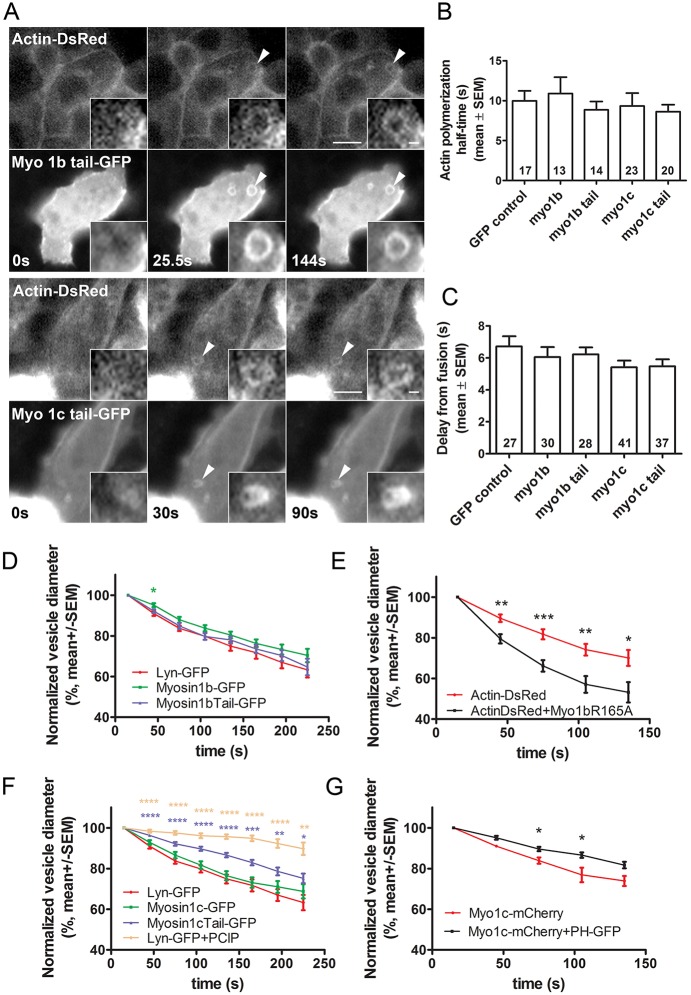
**Actin coat formation and compression in ATII cells transfected with Myo1b and Myo1c constructs.** (A) ATII cells co-transfected with actin–DsRed and Myo1b-tail–GFP (upper panel) or co-transfected with actin–DsRed and Myo1c-tail–GFP (lower panel). Myo1b-tail–GFP and Myo1c-tail–GFP translocated to fused secretory vesicles, which became coated with actin. Time=0 indicates the last time point before fusion, arrowheads point at the fusing vesicle and the insets show the enlarged view of fusing lamellar bodies. Scale bars: 10 µm (main images); 1 µm (insets). (B) Mean±s.e.m. of the half-time for actin polymerization on fused vesicles in ATII cells transfected with actin-DsRed and co-transfected with GFP or GFP-tagged Myo1b or Myo1c constructs. The numbers in the columns indicate the number of actin coats. No significant difference in the half-times for actin polymerization could be observed between cells transfected with GFP control and myosin constructs (two-tailed Mann–Whitney test). (C) Mean±s.e.m. of the delay from the fusion pore opening to the start of actin coat formation in ATII cells transfected with actin–DsRed and co-transfected with GFP-tagged Myo1b or Myo1c constructs. The numbers in the columns indicate the number of actin coats. No significant difference of delay from fusion for actin polymerization could be observed between cells transfected with GFP control and myosin constructs (two-tailed Mann–Whitney test). (D) Normalized vesicle diameter decrease (mean±s.e.m.) after fusion in ATII cells transfected with Lyn–GFP, Myo1b–GFP or Myo1b-tail–GFP (*n*=18, 31 and 44, respectively). A slower compression rate was observed in cells transfected with full-length Myo1b–GFP compared to Lyn–GFP. *P<0.05 (two-tailed *t*-test). (E) Normalized vesicle diameter decrease (mean±s.e.m.) after fusion in ATII cells transfected with actin–DsRed (control) or co-transfected with actin–DsRed and Myo1b-R165A–GFP. Fused vesicles in cells co-transfected with actin–DsRed and Myo1b-R165A–GFP (*n*=15) compressed significantly faster than control vesicles in cells transfected with actin–DsRed alone (*n*=16). *P<0.05, **P<0.01, ***P<0.001 (two-tailed *t*-test). (F) Normalized vesicle diameter decrease (mean±s.e.m.) after fusion in ATII cells transfected with Lyn–GFP, Myo1c–GFP, Myo1c-tail–GFP or Lyn–GFP treated with 10 µM PClP (*n*=18, 43, 34 and 12, respectively). Fused vesicles in cells transfected with Myo1c-tail–GFP or treated with 10 µM PClP inhibitor compressed significantly slower than vesicles in cells transfected with the control construct Lyn–GFP. *P<0.05, **P<0.01, ***P<0.01, ****P<0.0001 (two-tailed *t*-test). (G) Normalized vesicle diameter decrease (mean±s.e.m.) after fusion in ATII cells transfected with Myo1c–mCherry (*n*=5) and in cells co-transfected with Myo1c–mCherry and PH–GFP (*n*=29). The vesicle compression was slower in cells co-transfected with Myo1c–mCherry and PH–GFP; however, the differences were significant only at 75 s and 105 s. *P<0.05 (two-tailed *t*-test).

In addition to investigating the effect of dominant-negative Myo1b tail or Myo1c tail constructs on the percentage of responding cells, we also investigated their effect on actin coating during the post-fusion phase of lamellar body exocytosis. Transfection with dominant-negative Myo1b-tail–GFP or Myo1c-tail–GFP did not inhibit actin coat formation (Fig. 6A). The half-time of the actin coat polymerization in cells transfected with Myo1b-tail–GFP or Myo1c-tail–GFP was not significantly different from the half-time in cells transfected with wt Myo1b–GFP or wt Myo1c–GFP or from cells expressing control GFP construct (Fig. 6B). In addition, transfection with tail domains of Myo1c or Myo1b constructs had no effect on the delay from fusion to actin coat formation (Fig. 6C). Inhibition of Myo1 with PClP inhibitor also did not inhibit actin coats (Fig. S3).

Because the inhibition of Myo1b and Myo1c had no obvious effect on actin coat formation, we tested the hypothesis that the disruption of their function as a linker between actin coat and vesicle membrane might influence actin coat compression. The compression of fused vesicles in cells transfected with Myo1b-tail–GFP (measured as a decrease in vesicle diameter) was not significantly different from compression in cells transfected with the control construct Lyn–GFP. Vesicle compression in cells transfected with full-length Myo1b–GFP was slower than in control cells; however, the differences only reached significance at 45 s after fusion (*P*=0.01; Fig. 6D). Interestingly, fused vesicles in cells co-transfected with Myo1b-R165A–GFP and actin–DsRed compressed significantly faster than vesicles in control cells, which were transfected only with actin–DsRed (Fig. 6E). In contrast, vesicle compression was significantly reduced in cells transfected with the dominant-negative Myo1c tail (Fig. 6F). Pharmacological inhibition of Myo1 with PClP had an even stronger effect on the vesicle compression rate, as vesicles only compressed to 89.7% of the initial vesicle diameter in PClP-treated cells compared to 68.8% in Lyn–GFP-transfected cells (t=225 s; Fig. 6F). In addition, we measured the compression of vesicles in cells co-transfected with Myo1c–mCherry and PH–GFP to test for possible inhibitory effects of PH–GFP expression due to the binding of both constructs on PIP_2_. The vesicle compression in cells co-expressing both constructs was slightly reduced compared to cells transfected with Myo1c–mCherry alone and the differences were significant at time points 75 and 105 s after fusion (*P*=0.03 and *P*=0.01; Fig. 6G).

## DISCUSSION

We showed previously that compression of actin coats on fused secretory vesicles in ATII cells is necessary for surfactant extrusion (Miklavc et al., 2012). The actin coat compresses the fused vesicle by the combined action of myosin-II- and cofilin-mediated depolymerization of actin fibers followed by α-actinin-mediated cross-linking (Miklavc et al., 2015). Although we could identify Rho GTPases and nucleation factors of the formin family as initiators of actin coat polymerization on fused lamellar bodies (Miklavc et al., 2012), the mechanisms of actin coat attachment to the vesicle membrane are not clear. In this work, we demonstrate that Myo1b and Myo1c translocate to the membrane of fused secretory vesicles in ATII cells and that their inhibition influences the compression of actin coat. In addition, inhibition of Myo1c also reduces exocytosis in ATII cells, suggesting that the Myo1 family can play a role in several stages of surfactant exocytosis.

Myo1 family members attach actin cytoskeleton to membranes (Barylko et al., 2005; Coudrier and Almeida, 2011; Laakso et al., 2008; McConnell and Tyska, 2010) and have been suggested to play a role in several stages of secretory process (Bond et al., 2011; Woolner and Bement, 2009). Myo1c couples the actin coat to the membrane of fused vesicles in oocytes (Sokac et al., 2006). The translocation of Myo1c tail domain to the fused lamellar bodies suggests that the tail domain alone is sufficient for translocation to fused vesicles, supporting previously published observations (Hokanson et al., 2006; Sokac et al., 2006). Similarities in translocation kinetics of full-length Myo1c, the Myo1c tail or PH-PLCδ to lamellar bodies suggest that Myo1c translocation to fused vesicles is PIP_2_ dependent. Mutation of the basic residues in the PH domain (K892A and R903A), which prevent Myo1c binding to PIP_2_ lipids (Hokanson et al., 2006), completely abolished translocation of Myo1c to the lamellar body membrane after fusion.

Myo1b has been previously shown to localize to endosomal compartments as well as to PIP_2_-rich membrane regions (Komaba and Coluccio, 2010; Raposo et al., 1999; Ruppert et al., 1995) and its association with actin coats on fused vesicles had not been previously described. Sequence similarities between the Myo1c and Myo1b PH domain suggested that they would have similar lipid-binding properties (Hokanson et al., 2006). Although in ATII cells, the half-time of Myo1b tail translocation to fused lamellar bodies was not significantly different from the translocation of the Myo1c or Myo1c tail domain, the translocation of full-length Myo1b to the vesicle membrane was significantly slower. This is consistent with previous observations (Mazerik et al., 2014), where single-molecule total internal reflection fluorescence (TIRF) microscopy showed reduced mobility of full-length Myo1b in the plasma membrane. FRAP experiments in ATII cells showed slower diffusion of Myo1b in the plane of the plasma membrane compared to Myo1c or the control construct Lyn–GFP. In addition, the ATP-hydrolysis-deficient Myo1b R165A mutant translocated to the fused vesicles significantly faster than the wt Myo1b, and its translocation was transient, suggesting that actin-binding head domain might be involved in the retention of Myo1b at the membrane of fused vesicles.

Full-length Myo1b translocated to the vesicle membrane with similar kinetics to Lyn–GFP, which is inserted in the inner leaflet of the plasma membrane by myristyl and palmityl anchors (Kovárová et al., 2001). Although slow translocation of full-length Myo1b to the secretory vesicles after fusion might be explained by its actin binding, as discussed above, the equally slow translocation of Lyn–GFP cannot be explained in this way. FRAP experiments suggest that mobility of Lyn–GFP is comparable to the mobility of Myo1c and Myo1c tail constructs, which is consistent with reports on fast diffusion of Lyn–GFP in the plane of the plasma membrane (Schmidt and Nichols, 2004). It is therefore possible that the rapid translocation of full-length Myo1c and Myo1c tail to the fused vesicles is due to the recruitment of Myo1 from the cytosol. Alternatively, the fusion pore structure might represent a barrier for lateral diffusion of membrane proteins with lipid anchors.

Cytosolic Ca^2+^ plays an important role for surfactant secretion. The increase in intracellular Ca^2+^ concentration caused by cell stretch or extracellular ATP are the most potent physiological stimuli leading to lamellar body exocytosis (Dietl et al., 2010, 2012) and can also contribute to the widening of the fusion pore (Miklavc et al., 2011). The motor activity and membrane-binding properties of Myo1 depend on the cytoplasmic Ca^2+^ concentration (Greenberg and Ostap, 2013; McConnell and Tyska, 2010; Tang et al., 2002) and it is therefore possible that changes in Ca^2+^ concentration during exocytosis in ATII cells might influence Myo1 translocation to the secretory vesicles as well as its function. Ca^2+^ binding changes the Myo1 force generation capability (Lin et al., 2005; Swanljung-Collins and Collins, 1991; Zhu et al., 1996) and has also been suggested to promote membrane attachment (Cyr et al., 2002; Swanljung-Collins and Collins, 1992). In contrast, elevation of intracellular Ca^2+^ concentration by ionomycin in HEK cells caused the translocation of the Myo1c tail domain from the plasma membrane to the cytosol (Hokanson and Ostap, 2006). Our findings in ATII cells also suggest that Ca^2+^ elevation does not promote membrane binding. Addition of ionomycin resulted in a marked translocation of Myo1c and Myo1b from the plasma membrane to the cytosol. Stimulation with PMA had no effect on Myo1b translocation. However, it caused a small but significant translocation of Myo1c from the plasma membrane to the cytosol. This observation might be due to the direct effect of PMA on the decrease of PIP_2_ concentration on the plasma membrane (Zeng et al., 2003), which might affect Myo1c more strongly than Myo1b. The translocation to the membrane of fused vesicles was slower after ionomycin stimulation compared to PMA stimulation for both the Myo1b and Myo1c constructs. Interestingly, the translocation half-times after ATP and PMA stimulation were not significantly different. This might be explained by the transient Ca^2+^ increase after ATP stimulation versus prolonged cell fusion response.

Myo1b and Myo1c translocated to the fused secretory vesicles after fusion pore opening, which suggests that their main function might be during the post-fusion phase of exocytosis. We did not find significant differences in actin coat polymerization when we compared half-times of actin polymerization and the delay between fusion and the start of actin coat formation after Myo1b or Myo1c inhibition. This suggests that Myo1b and Myo1c likely do not play a major role in actin coat formation. However, our results suggest that Myo1b and Myo1c might be necessary for the normal coat compression. In oocytes, uncoupling of the actin coat from the vesicle membrane by transfecting cells with the Myo1c tail resulted in inhibition of coat compression (Sokac et al., 2006). We could confirm this observation in ATII cells. Interestingly, although the transfection of cells with the Myo1b tail had no effect on vesicle compression, we observed a marked increase in the vesicle compression rate after transfecting the cells with the Myo1b R165A mutant. Conversely, overexpression of wt Myo1b resulted in a slight decrease in vesicle compression rate. These observations suggests that Myo1b might play a role of a tension sensitive ‘anchor’ for the vesicle compression as suggested by biophysical properties of Myo1b (Laakso et al., 2008, 2010). PClP, an allosteric inhibitor that reversibly binds near the actin-binding region on the myosin motor domain has been shown to be specific for Myo1 family, and inhibits Myo1c as well as Myo1b (Chinthalapudi et al., 2011; Martin et al., 2009). We observed that PC1P treatment decreased the rate of vesicle compression without affecting actin coat formation, similar to genetic inhibition of Myo1c. This might be due to a higher expression of Myo1c in ATII cells or to a more notable physiological role of Myo1c in actin coat compression as compared to Myo1b. A similar observation was made in HeLa cells, where PClP inhibition resulted in identical changes in lysosomal morphology as described for the small interfering RNA (siRNA)-mediated knockdown of Myo1c (Chinthalapudi et al., 2011).

Myo1c was not only reported to connect the actin coat to the membrane of fusing vesicles but also to facilitate vesicle fusion with the plasma membrane. In adipocytes and skeletal muscle cells, Myo1c inhibition leads to a decreased exocytosis of GLUT4-containing vesicles after insulin stimulation (Bose et al., 2002, 2004; Toyoda et al., 2011) and in HeLa cells to decreased incorporation of GPI-linked proteins in the plasma membrane (Brandstaetter et al., 2012). TIRF experiments showed that Myo1c tethers GLUT4 vesicles to the plasma membrane and the absence of this tethering inhibits exocytosis (Boguslavsky et al., 2012). It is not yet clear whether Myo1c promotes lamellar body exocytosis in ATII cells through the same tethering mechanism. However, it is more likely that the function of Myo1c is in cortical actin rearrangement or lamellar body tethering to the plasma membrane than in active vesicular transport. Although a proteomics study on isolated lamellar bodies has suggested that there are Myo1c as well as Myo1b molecules associated with lamellar bodies (Ridsdale et al., 2011), we could observe a strong translocation of Myo1c and Myo1b to lamellar body membrane only after fusion, whereas their presence on non-fused lamellar bodies was not detectable by conventional fluorescence microscopy.

Together, these data indicate that Myo1c and Myo1b translocate to secretory vesicles in ATII cells after fusion. The translocation of Myo1c to the vesicle membrane closely resembled the translocation of PH-PLCδ, whereas the translocation of Myo1b was markedly slower. The inhibition of Myo1c reduced secretory vesicle exocytosis and slowed down actin coat compression. In contrast, Myo1b R165A induced an increase in actin coat compression rate, suggesting that several mechanisms are necessary for efficient actin coat compression.

## MATERIALS AND METHODS

### Cell isolation and transfection

ATII cells were isolated from the lungs of male Sprague-Dawley rats, aged 3–4 months, according to the procedure of Dobbs et al. (1986) and cultured as recently described (Miklavc et al., 2010). Rats were kept at the central animal facility of the Ulm University according to guidelines for ethical care of animals. All experiments in this study were approved by the Regierungspräsidium Tübingen, Germany. Isolated cells were used for experiments for up to 48 h after isolation because longer cell culture times result in rapid loss of ATII cell characteristics (Diglio and Kikkawa, 1977; Mason and Dobbs, 1980). ATII cells were transfected by electroporation or by adenoviral vectors as recently described (Miklavc et al., 2015).

### Plasmids and adenoviral vectors

Plasmids expressing Myo1c–GFP, Myo1c-tail–GFP, Myo1c-R903A–GFP and Myo1c-K892A–GFP were a kind gift from E. Michael Ostap (University of Pennsylvania, Philadelphia, USA) (Hokanson et al., 2006); Myo1b–GFP and Myo1b-tail–GFP were generously provided by Evelyn Coudrier (Institut Curie, Paris, France) (Raposo et al., 1999) and PH-GFP adenovirus was kindly provided by Ora Weisz (University of Pittsburgh, Pittsburgh, USA) (Weixel et al., 2007). Myo1b-R165A–GFP was created with the Infusion system (Clontech, TakaraBio, France). Myo1c–mCherry was made by cloning PCR-amplified mCherry from the pmCherry-N1 vector (Clontech, TakaraBio, France) in Myo1c–GFP using *Age*I and *Not*I restriction sites. Myo1b–mCherry was made by cloning PCR-amplified mCherry from pmCherry-N1 vector (Clontech, TakaraBio, France) in Myo1b–GFP using *Nhe*I and *Xho*I restriction sites. Adenoviruses expressing actin–GFP and actin–DsRed and plasmid for Lyn–GFP were recently described (Frick et al., 2007; Miklavc et al., 2009, 2012). As control GFP plasmid we used pmax GFP™ (Lonza, Germany).

### Experimental conditions

For all experiments, ATII cells were kept in bath solution (in mM: 140 NaCl, 5 KCl, 1 MgCl_2_, 2 CaCl_2_, 5 glucose, 10 Hepes; pH 7.4). Cells were stimulated for secretion with 100 μM ATP (Sigma, Schnelldorf, Germany), 300 nM PMA, or 3 µM ionomycin. Fusions were detected with LysoTracker Red (10 nM, 10 min incubation) or LysoTracker Blue (100 nM, 20 min) (Haller et al., 1998). For pharmacological inhibition of Myo1, ATII cells were preincubated with 10 µM PClP inhibitor for 2 h. Fluorescent dyes were purchased from Molecular Probes (Life Technologies, Darmstadt, Germany).

### Semi-quantitative RT-PCR

Semi-quantitative RT-PCR was performed on 0.8–1.3 µg RNA with SuperScript VILO synthesis kit and QuantiTect primer assays (Quiagen, Hilden, Germany) on a realplex2 mastercycler (Eppendorf, Hamburg, Germany) as described in detail previously (Miklavc et al., 2015).

### Western blot analysis

Western blot was performed on freshly isolated ATII cells or ATII cells after 2 days of culture as described in detail previously (Miklavc et al., 2011). Primary antibody against Myo1c purchased from Abcam (catalogue number ab51261, lot number GR150630-3; Cambridge, UK) and primary antibody against Myo1b purchased from Novus biologicals (catalogue number NBP1-87739, lot number A39461; Littleton, USA) were used in concentrations suggested by the manufacturer (1:1000 for Myo1c and 1:500 for Myo1b). The manufacturer verified antibody specificity.

### Immunofluorescence

Primary antibody against ABCa3 was purchased from Abcam (catalogue number ab24751, lot number GR133811-2). Fluorescently labeled secondary antibodies and Alexa-Fluor-568-conjugated phalloidin were purchased from Molecular Probes (Life Technologies, Darmstadt, Germany). We used primary antibody dilutions suggested by the manufacturer (1:50 for anti-Myo1b and -Myo1c antibodies, 1:300 for anti-ABCa3 antibody). Images were taken on Leica TCS SP5 confocal microscope (Leica, Wetzlar, Germany) using a 63× objective (Leica HCX PL APO lambda blue 63.0×1.40 OIL UV). Images for the green (Alexa Fluor 488), red (Alexa Fluor 568) and far red (Alexa Fluor 647) channels were taken sequentially using appropriate excitation and emission settings.

### Fluorescence imaging

Fluorescence imaging experiments on living cells were performed on an iMic digital microscope (Till Photonics, Gräfelfing, Germany) as described previously (Miklavc et al., 2015). FRAP experiments were performed on an iMic microscope using a green laser at 95% intensity to bleach a circular region of interest (diameter=4 µm). DiI-stained cells were imaged on a Cell Observer microscope (Visitron, Puchheim, Germany) with 40× oil immersion objective and MetaMorph acquisition software (Molecular Devices, Sunnyvale, USA).

### Image analysis and data presentation

Images were analyzed using Fiji (NIH, Bethesda, USA). GraphPad Prism 5 (GraphPad Software, La Jolla, USA) was used for curve fitting and graph design. Lamellar body compression after fusion was analyzed by measuring the vesicle perimeter at indicated time points after fusion. A circular region of interest was set around the fusing lamellar body to determine the onset of lamellar body fusion by measuring LysoTracker fluorescence or to measure the translocation of fluorescently labeled constructs (Miklavc et al., 2012, 2015). Figures were prepared with Adobe Photoshop CS2 (Adobe, San Jose, USA).

### Statistical analysis

We used Excel (Microsoft, Redmond, USA) and GraphPad Prism 5 (GraphPad Software, La Jolla, USA) for statistical analysis. Unless stated otherwise, *n* indicates the number of fused vesicles where fluorescence changes were measured and was set to ≥5. The fusions were recorded in 4–21 independent experiments on ATII cells from 2–7 cell isolations. Only vesicles where the fluorescence signal to noise ratio was sufficiently high and where the whole secretory process could be monitored were used for analysis (predefined). Unless stated otherwise data are presented as mean, the s.e.m. was used to estimate the variation within data groups (indicated for every data set) and two tailed *t*-test was used for statistical comparison. The D'Agostino and Pearson omnibus normality test was used to estimate the data distribution.
